# Tissue Remedies*Bureau Materia Medica, I. H. A., June, 1887.

**Published:** 1888-09

**Authors:** Eugene B. Nash

**Affiliations:** Cortland, N. Y.


### TISSUE REMEDIES.*

EUGENE B. NASH, M. D., CORTLAND, N. Y.

In a lecture on Colocynthis, Dr. J. T. Kent speaks by way of comparison of Mag-phos. as an excellent remedy in colic. I do not remember his exact words, but do remember that, while both of these remedies have the crampy, doubling up pains, for which Coloc. has so long and often been found promptly remedial, Mag-phos. has also, in almost as marked degree as Ars-alb. *relief from heat.*

Not only is this so in Mag-phos. but also in some other neuralgic pains and affections for which Mag-phos. has often been found efficacious. So if Coloc. fails me in neuralgias, where the

---

* Bureau Materia Medica, I. H. A., June, 1887.

pains are cramping, I look sharp for the amelioration from heat and if it appears, Mag-phos. generally *cures.*

Not long ago I was called to a case of facial neuralgia, of long standing, which had been treated by a regular with anodynes, large doses of Belladonna being the last one used, in such doses that the muscles of accommodation in the eye were so paralyzed that the patient could not recognize the friends taking care of her. Upon examining her case, I found that the only way in which she could get any, even momentary relief, was by hot applications to the painful nerve. Here was a good opportunity to try Mag-phos. I gave it in the twelfth trit., a small powder dissolved in water, and teaspoonful doses at intervals of two or three hours. A few doses soon relieved her of the pain and in a few days more she had recovered from the weakness consequent upon such long suffering.

Now what of the tissue remedies of Schussler? Shall we refuse to use them because they have not been proven? I say yes, if we can find our remedy among drugs that *have been* proven; if not, use them, especially if their clinical use in the *potentized form* has proved repeatedly successful. Many of the most reliable symptoms in our *Materia Medica* have been discovered *ab usu in morbis;* like that of Puls. with which I perfectly relieved an old man suffering from enlarged prostate (after he had been aspirated twice to empty the bladder) on the symptom, "after micturition, even a few drops, spasmodic pains in the neck of the bladder, extending to the thighs." I was led to it by Dr. Lippe, in his excellent article on prostatic diseases in the Hom. Phys. Dig. and Caust. have served me equally well when indicated. This valuable symptom is now in Hering's *Condensed Materia Medica* but is not found in Lippe, Allen, or Cowperthwaite, and has never, so far as I know, been produced in proving. A diamond discovered by *accident* is as truly a diamond as though you had been *hunting* for it; so these clinical gems in the *Materia Medica* are no less gems because we have not a full proving of the drug to which they belong. This is especially so if they appear during the use of the *potentized* drug. If a symptom or diseased condition is cured or removed with a remedy from the thirtieth or even the twelfth dil. upward and that *repeatedly*, I "chalk it down;" it is entitled to my confidence. I believe it almost impossible to *cure* with the thirtieth potency, or even to *remove* any symptom or set of symptoms to which it is *not* homœopathic. Or in other words the anti-pathic remedy in such a potency will produce no curative effect at all. So if a cure *does* follow the administration of such a potency the remedy must have been homœopathic to the symptoms, and a *thorough proving* of the remedy will corroborate it. One thing must be remembered, viz.: that while cures must verify provings, provings (if carried far enough) must also verify cures that are made accidentally. Now am I advocating empiricism? Not a bit of it. Let me be understood; head first is the way a child *should* be born, but if he comes breech first, it is a child all the same. Provings first, and cures afterward is the *right* way, but if cures come first, accept them, and make the *provings* afterward. Such cures are, sometimes, wonderful leaders to most valuable therapeutic agents.

Now in regard to these tissue remedies. Let any honest investigator study the symptoms of Ferrum as laid down in the Homœopathic *Materia Medica*, its vertigo, congestion to head, penetrating hammering pains, red face, epistaxis, etc. Then study it in its action on the lungs; note the oppression, hæmoptysis with bright red blood, interscapular pains; together with the fact that Hempel mentions, that those who live in the vicinity of iron springs and drink the water are subject to local congestions, pulmonary phthisis, spitting of blood, etc. Add to all this the well-known action of Phos. upon the lungs, and then judge whether the biochemic theory, or the *homœopathic law* for applying Ferr-phos. in local congestions of the head, lungs, or any other part lays best claim to our confidence as guide. Calc-phos., Natr-sul., Nat-m., and Silicea are well proven, and have been long used in many of the diseases for which Schussler recommends them. No doubt they affect the tissues; so does every remedy, vegetable, animal, or mineral, which changes diseased into healthy action or *vice versa*. But whether they cure by virtue of supplying any lack of chemical elements in the tissues, rather than by correcting a condition of the system, which in *health*, is able to select these elements necessary to its well being is very doubtful to say the least. Every true homœopath knows that the lack of bone development for which Calc-phos is remedial, is readily corrected by the use of this remedy in the thirtieth, two hundredth, or even higher potencies. How much of the lacking chemical element would be supplied in a month, provided the child took a grain of the thirtieth three times a day let the Milwaukee Bureau answer.

Well, time and research will bring many facts to light that will corroborate the truth of "similia similibus," etc., and one of them will be that the tissue remedies of Schussler are *wonderful remedies;* another that they will cure in potentized form, all diseases *to which they are homœopathic and no others.*

DISCUSSION. Dr. Lippe—It strikes me our dear friend is going back a century. You will remember the conversation with Samuel Hahnemann in regard to a case where a remedy was applied for the cure of acute intermittent fever. The question was asked, under what conditions the provings were made, and Hahnemann replied, "Cullen made his cures before, and Hahnemann proved afterward," and since that day the language has been reversed as far as the proving of medicine and making of cures. Of course, Cullen did not understand it, but it is a law of cure and you have it to-day—the same thing over again. I don't think we have any need to go back into the last century. *You will prove your medicine first and cure afterward.*

Dr. Nash—That is what I said; that is what I claim in the paper: *Prove first and cure afterward.* And I want that clearly understood. If Cactus is given a patient, and is homœopathic to him, it will cure him whether it has been proved or not. That is the idea. Of course, it is better to have the proving first than to have the clinical symptoms. These clinical symptoms are valuable after they are proved; provided those symptoms were never produced. I do not defend that kind of way of doing business, understand that. I advocate the proving of a remedy first. But if we do discover, as we say, that a symptom is produced by a remedy, and that remedy when repeated, produced the same symptom again, and when used for the purpose of curing, it does cure, then don't reject it because it is clinical.

Dr. Wesselhœft—I believe we have three indications for the use of this remedy, and they are symptoms not derived from provings, but observed upon the sick. We have a tripod and it will stand. I made a cure with Mag-phos. very similar to the one reported by Dr. Nash, and it is one I think could never have been made without the knowledge of the characteristics of this drug. To me it was an astonishing cure; and the city of Eastport sent me a large delegation of patients in consequence of it. The subject was an old lady, sixty-six years of age, whom I had never seen, and when I did see her six or seven years later, I wondered that a cure of any disease should be possible in a subject so attenuated, anæmic, and lacking in everything which might show a promise of favorable reaction to even the most specific remedy. The pain was supra-orbital, *right-sided*, sharp, *intermittent*, and relieved by *warm* applications. It had lasted, with short intervals of relief, for three years. She received at intervals of four or five weeks, single doses of Mag-phos.$^{cm}$ for three months. She has remained practically free from pain ever since. Mag-phos. has its pains on the right side; they are intermittent and relieved by warmth. This is true of the facial pains as well as the ovarian and uterine pains. I was anxious to ascertain from Dr. Nash if his patient's pains occurred on the right side, but unfortunately he did not remember.

The pains of Mag-phos. are similar to those of Pulsatilla, but the latter remedy has intolerance of, and aggravation from, external warmth.

It is a remedy that we should prove as soon as possible, and it was my intention to offer it for proving this year. I was, however, so impressed with a cure by Dulcamara for which I could find no reason from the symptoms as far as at present known, that I was anxious this drug should be more fully proven upon women. Of all the so-called "tissue remedies" (I refer only to those which have as yet no provings), Mag-phos. is the only one of which we know that it has at least three characteristics—and if these three are present in a case, it will cure.

Dr. Baylies—-On which side did you say the pain was?

Dr. Wesselhœft—The so-called ovarian pains were upon the right side. In this case the pains were on the left side and in the face. I think it a very peculiar case. You will recollect that the pain is on the right side in facial neuralgia.

Dr. Wells—I heard a part of the paper of my friend, and believe I heard enough to get this idea, that he was to give a certain degree of value, as I understand, to a remedy which had cured but had not been proven. These cures incidentally, if you please, have their value. I do not think there will be any difference of opinion in the Association as to that. They cannot, of course, rank with symptoms which have been ascertained by proving, and there will be no difference of opinion on this point. There is a different degree of importance attached to them. But a marked cure made by a drug like that, of symptoms which it has cured, and are not found in a proven drug, is a guide-board pointing in the right direction. A matter to be observed, and I think my friend is perfectly justified, in calling attention to the importance of them—partly proven, proven by clinical use. I think that if we get a baby by the heels that we have a baby, and it is just as good a baby afterward as if it was born by the head.

Dr. H. C. Allen—Mr. President, I was very much pleased with Dr. Nash's paper. I was afraid, however, that when he started out, he was going to advocate heel delivery instead of the natural form.

Dr. Nash—No, sir.

Dr. H. C. Allen—Like some other acrobats, he comes right side up after all. I should like to affirm what he has said in regard to some of the characteristic pains of Magnesia phosphorica. I had last winter a patient that suffered intolerably with right-sided chest pains. He was a pastor. I had relieved him a number of times, but the relief would only be temporary until a severe northwest storm set in; then they would come back—those chest pains. I found a few of his symptoms under Magnesia, and I decided, taking all into consideration, to give him a few doses of the thirtieth trituration of Magnesia-phos. I think that was a victory. He has been blowing my horn all over the city ever since. He has not had an ache or pain in that right side. That confirms what Dr. Wesselhœft said about the darting, shooting, forcing, tearing character of the neuralgic pains. These were not intermittent any further than the storm was concerned in affecting the right side. It was a northeast storm that came off one of our fresh-water lakes, the worst kind of a storm for rheumatic and neuralgic troubles. I may say, for Dr. Wesselhœft's gratification, that I have two provers at work on Magnesia-phos., and they have been at it for about two months.

Dr. Nash—I would say, to close this discussion, that I thought some might be lead into the habit of prescribing remedies on the idea that they might possibly hit it. The idea was not of that kind. I do not advocate any such thing by any means. I hunt for my remedy if it is in the *Materia Medica.* If I find some other man, Dr. Wells, Dr. Lippe, or any other man, has cured several times with a remedy, it may be that it came by accident first—that is the next thing I go for—one of the next things.

Dr. Brown—For four years I have used Schussler's preparations if I could not get indications for other remedies. First hunt by the law of similars—sometimes you will come across poisonous conditions produced by old-school physicians. In those cases the provings will not be of much value. In order to have a good proving we have to follow homœopathic law. Hunt for your knowledge. That is the idea; and when you find it you can use it. Take advantage of the circumstances, as any man would in using hot water, cold applications, or exercise, or any other remedy that is used because we have proved it. We have found it was good for the circulation. I have often made cures entirely in that way without giving any homœopathic remedy. If the patient has been indulging in treatment by the old-school practitioners, and is filled up with Quinine, then you must treat them accordingly, and I am sorry to say that some men who are called homœopaths, but who would not be allowed to enter this Association because they use Quinine, have it on their table, and use Morphia, because they are too lazy to select the right remedy. If there are such in our school now, they do not follow after me.

